# A Norwegian 15D value algorithm: proposing a new procedure to estimate 15D value algorithms

**DOI:** 10.1007/s11136-018-2043-9

**Published:** 2018-11-30

**Authors:** Yvonne Anne Michel, Liv Ariane Augestad, Mathias Barra, Kim Rand

**Affiliations:** 1 Department of Health Management and Health Economics, Medical Faculty, University of Oslo, Postboks 1089, Blindern, 0318 Oslo, Norway; 2 The Health Services Research Unit - HØKH, Akershus University Hospital, Lørenskog, Norway

**Keywords:** Health-related quality of life, 15D, Visual analogue scale, Value algorithm

## Abstract

**Purpose:**

So far there is no Norwegian value algorithm to inform healthcare decision making. The 15D health state values estimated with the original 15D valuation procedure tend to be higher than the values of other generic preference-based health-related quality of life (HRQoL) instruments. The main purpose of this study was to use a new 15D valuation procedure to estimate Norwegian 15D health state values and to explore their empirical performance.

**Methods:**

The visual analogue scale was used to collect 15D valuation data in a representative sample of the Norwegian general population. The new procedure used fewer valuation tasks and anchored the 15D health state values in an empirically assessed range. The Norwegian 15D health state values were compared to the values of five HRQoL instruments which were provided by Norwegian residents belonging to seven disease groups and a healthy population.

**Results:**

The Norwegian 15D health state values ranged from 1 to − 0.52. Compared to 15D health state values estimated with the original procedure, the Norwegian 15D health state values were lower and more in line with values of other HRQoL instruments.

**Conclusions:**

The new 15D valuation procedure is simpler, links the 15D health state values better to the requirements of the QALY model, and provides an empirically-based range. We recommend using the new valuation procedure in future 15D valuation studies, and the Norwegian health state values for use in 15D-based health economic analyses in Norway.

## Introduction

The 15D is a generic preference-based health-related quality of life (HRQoL) instrument that can be used to provide preference-weights for quality-adjusted life-year (QALY) calculations when paired with valuation data [1, 2]. It is used internationally [1] and was recently a part of a large multi-instrument comparison study (MIC) [3, 4]. The 15D assesses HRQoL via a descriptive system with 15 dimensions, covering physical, mental, and social aspects of health [2, 5]. Originally Finnish, the 15D has been translated into 30 languages, including Norwegian [1, 6]. 15D valuation studies have been conducted in Finland [7] and Denmark [8]. These studies used the same study design involving three valuation tasks, based on the visual analogue scale (VAS), and were carried out through postal administration [7, 8]. In this study, we addressed a selection of problems known to be related to the original 15D valuation system. We reviewed the original 15D valuation tasks with a focus on how the information they provide is combined to estimate 15D algorithm values. An earlier study suggested that the way information from the three original 15D valuation tasks is combined increases the likelihood of larger error terms which makes the link between the VAS tasks and the health state values less transparent [9]. We propose a new 15D value algorithm estimation procedure that uses information from only one of the original valuation tasks [9] and anchors the worst possible health state in an empirically assessed value.

There is no *value algorithm* (or tariff, or value set) based on preferences of the Norwegian general adult population for any generic preference-based HRQoL instrument. It is the main aim of this study to use a new 15D valuation procedure to estimate a Norwegian 15D value algorithm. Further, we compared the resulting Norwegian 15D health state values with corresponding values of other generic preference-based instruments collected in seven disease groups and a healthy sample.

## Methods

### The 15D instrument

The 15D health state space comprises 15 dimensions, each with five levels of function [2, 5]. As such, a 15D health state can be represented as a vector $\mathbf{l}=\left( {{l_1}, \ldots ,{l_{15}}} \right)$, where each ${l_j}$ specifies the level of function on dimension $j$. The 15D has a large number of dimensions and levels and its full health state space contains 5^15^ health states. It is cognitively challenging to value 15D health states, consisting of 15 attributes and unfeasible to directly evaluate a representative set of 15D health states [7]. Instead, each level of function of the 15D descriptive system was valued separately with VAS-based valuation tasks and simplifying assumptions from multi-attribute utility theory were used to derive 15D health state values in the original 15D algorithm estimation procedure [7, 10].

**Fig. 1:**
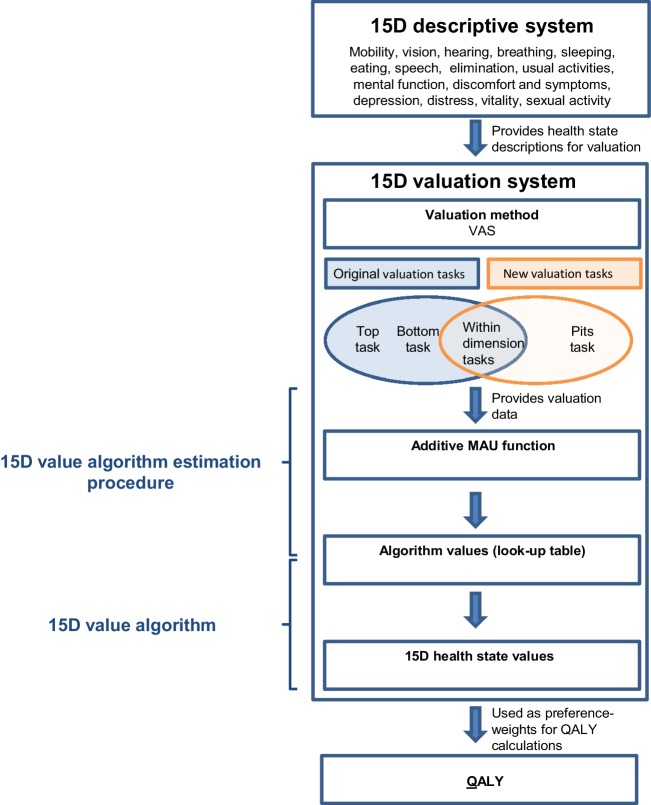
Overview of the 15D valuation system. *VAS* visual analogue scale, *MAU* multi-attribute utility, *QALY* quality-adjusted life years

A *value algorithm estimation procedure* details the steps needed in order to convert valuation data from valuation tasks into a value algorithm (Fig. 1). A 15D *value algorithm* consists of a look-up table with algorithm values which allow estimating a value to each 15D health state (Fig. 1). As the 15D health state space is considerably large, it is common to present a look-up table which allows to estimate 15D health state values by adding up 15 algorithm values (Fig. 1). We thus describe a procedure that converts valuation data, i.e., the participants’ responses to the VAS-based valuation tasks, into a value algorithm that yields health state values ${V_H}(\mathbf{l})$ for all 15D health states $\mathbf{l}$.

### The original 15D valuation tasks and the original value algorithm estimation procedure

The original 15D valuation procedure consisted of three VAS-based valuation tasks: the *top task*, the *bottom task*, and the *within dimension tasks* (Fig. 1). The top and the bottom tasks each used a single VAS on which respondents were asked to evaluate the best and the worst levels of all 15 dimensions. These tasks were intended to provide information about how important each dimension was perceived. In the 15 within dimension tasks, the respondents were asked to assign a VAS-score to each level of impaired function, referred to as L2 through L5, and “being dead” for each dimension separately, while L1 was fixed at 100 (Appendix 1). In the original procedure, the resulting level scores were multiplied with the importance weight for each level, which was “extrapolated linearly” [7] based on information from the top and bottom tasks [2]. A step-by-step description of the original procedure can be found in Appendix 3 in Michel et al. [9].

### The new 15D valuation tasks and the new value algorithm estimation procedure

The new 15D algorithm estimation procedure used information of the *within dimension tasks* and a *pits-task* (Fig. 1). While the within dimension tasks were identical with the tasks used in the original valuation procedure, the pits-task was only part of the new valuation procedure. Of the original three tasks, only information from the within dimension tasks was retained. Thereby problems from combining information from different valuation tasks were avoided, and the link between the VAS-scores and the resulting health state values has become more transparent [9]. The within dimension tasks provided relative VAS-scores for all levels. The pits-task provided an average score for the *worst possible 15D health state*, L5 on all 15 dimensions, that has been directly scored on a VAS together with “being dead” (Appendix 2). Besides the pits-task, no interactions between the 15D levels were assessed.

The QALY model assumes that preferences for health states can be expressed on a ratio-scale, where zero corresponds to “being dead” [11], where “being dead” is a proxy for health states without an intrinsic (QALY-) value, since “being dead” suspends time [12–14]. Using the pits-score’s relation to “being dead” and “perfect health” to determine the range of the 15D health state values provided a plausible link to the ratio-scale used in the QALY model.

The new value algorithm estimation procedure was performed on averaged within dimension VAS-scores that were weighted to match the general Norwegian population. Sixteen task-specific sets of weights have been estimated (one for each of the fifteen within dimension tasks, and one for the pits-task). Post-stratification weights have been applied (see Appendix 3 for information on which variables were used for post-stratification and a detailed description of the weighting procedure).

Health state values represent disutility values via the identity $~v~=~1 - u$. The new value algorithm estimation procedure yields a 15D health state value ${V_{H~}}(\mathbf{l})$ for a 15D health state ${\mathbf{l}} = \left( {l_{1} \cdots l_{{15}} } \right)~$ using the following functional form:

$${V_H}\left( \mathbf{l} \right)\mathop =\limits^{{{\text{def}}}} 1 - \frac{{{V_{Pits}}}}{{\mathop \sum \nolimits_{j} {S_{j,5}}}}~\mathop \sum \limits_{{1 \leqslant j \leqslant 15}} {S_{j,{l_j}}}=1 - ~\mathop \sum \limits_{{1 \leqslant j \leqslant 15}} \omega \times {S_{j,{l_j}}}=1 - \mathop \sum \limits_{{1 \leqslant j \leqslant 15}} {T_{j,{l_j}}}$$

- Step 1: For each dimension $j$ and level $i~$ (L2–L5 and “being dead”), compute the weighted means $\overline {s} _{i}^{j}$ over the respondents’ raw VAS-scores from the within dimension tasks (Table 6 in Appendix 4).
- Step 2: For each level $i$ (L2–L5), estimate the level’s relative scores $~{{S}_{j,i}}~$, within each dimension $~j$, as $~{\widehat {S}_{j,i}}~\mathop =\limits^{{{\text{def}}}} ~\frac{{100 - \overline {s} _{i}^{j}}}{{100 - \overline {s} _{{{\text{death}}}}^{j}}}$, resulting in within dimension disutility values (where ${\widehat {S}_{j,1}}=0$ since $\overline {s} _{1}^{j}=100~$ by definition, see Table 7 in Appendix 4).
- Step 3: Estimate the mean of the respondents’ empirically obtained scores for the pits-state as ${\widehat {V}_{{\text{Pits}}}}\mathop =\limits^{{{\text{def}}}} ~\frac{{100 - {{\overline {s} }_{{\text{Pits}}}}}}{{100 - {{\overline {s} }_{{\text{death}}}}}}$, resulting in one disutility value for the pits-state representing the estimated health state value-range.
- Step 4: Estimate the within dimension disutility table ${{T}_{j,i}}~$ (Table 1) by rescaling the level scores ${\widehat {S}_{j,i}}~$ by the rescaling factor $\widehat {\omega }\mathop =\limits^{{def}} \frac{{{{\widehat {V}}_{{\text{Pits}}}}}}{{\mathop \sum \nolimits_{j} {{\widehat {S}}_{j,5}}}}: {{\widehat {T}}_{j,i}} \mathop=\limits^{{def}} {\widehat {\omega }}\mathop \cdot {{\widehat {S}}_{j,i}}$. This normalizing constant ensures that the final range of health state utilities is bounded by 1 for “perfect health” and $1 - {\widehat {V}_{{\text{Pits}}}}$ for the pits-state.
  A 15D health state value ${V_{H~}}\left( \mathbf{l} \right)$ is a simple sum of fifteen of the disutility values presented in Table 1.

**Table 1:** The Norwegian 15D algorithm disutility values

	Mobility	Vision	Hearing	Breathing	Sleep	Eating	Speech	Elimination	Usual activities	Mental function	Discomfort	Depression	Distress	Vitality	Sexual activity
Level 2	0.0357	0.0300	0.0304	0.0367	0.0327	0.0392	0.0386	0.0388	0.0331	0.0413	0.0382	0.0390	0.0367	0.0369	0.0297
Level 3	0.0612	0.0625	0.0583	0.0612	0.0524	0.0721	0.0603	0.0729	0.0612	0.0687	0.0689	0.0625	0.0589	0.0569	0.0637
Level 4	0.0876	0.0839	0.0830	0.0825	0.0774	0.0952	0.0847	0.0915	0.0831	0.0894	0.0899	0.0878	0.0831	0.0805	0.0849
Level 5	0.1083	0.1012	0.0959	0.1008	0.0968	0.1061	0.0960	0.1038	0.1016	0.1022	0.1063	0.1039	0.0978	0.0976	0.0975

The Norwegian 15D algorithm values are presented as disutility values, which can be interpreted as the value loss that is associated with the respective level of function. Level 1 is associated with no value loss and corresponds to zero on all dimensions

We estimated a Norwegian 15D value algorithm using this new procedure. We present the range and the dimensions with the largest and the smallest disutility values. The original 15D algorithm estimation procedure was applied to the Norwegian valuation data in an earlier study [9] for comparison, and the resulting values were not recommended for use in healthcare decision making. We refer to the values resulting from the previous comparison as *original* 15D values, in contrast to the *new* 15D values estimated in the current study.

### The Norwegian valuation studies

The main *Norwegian 15D valuation study* was conducted in 2010 through the marked research firm TNS Gallup. The survey was self-completed and consisted of demographic variables, the 15D descriptive system (self-reported health), and the original 15D valuation tasks. Parallel Web and postal surveys were conducted. The postal survey was sent to a random sample of about 5000 postal addresses from the Norwegian National Population Registry. Participants received a prepaid response envelope. The Web sample was recruited by sending mails to 1936 individuals pre-registered in an online panel maintained by TNS Gallup (TNS-Gallup Panel). Several waves of emails were sent out to reach a responding sample of 1000+ individuals that resembled the Norwegian general population in terms of age, gender, educational level, and geographic distribution. Due to technical limitations in the software used for the Web data collection, the task presentation differed between the Web and postal sample (see Appendix 1 for details). For details about how the different survey versions were randomized and for the exclusion criteria applied in this study, see [9]. Sensitivity analyses for testing the influence of case exclusion on within dimension task scores and 15D algorithm values were performed and did not indicate any differences between the unselected and the selected sample [15].

An additional face-to-face data collection was conducted in 2015–2016 in order to directly assess the value associated with the worst possible 15D health state. We asked 120 members of the Norwegian general population to provide information on age and gender and to fill in the 15D descriptive system. Further, the participants performed two within dimension tasks to get familiar with the 15D valuation tasks. Finally, the pits-task was presented in which participants were instructed to score the worst possible 15D health state and “being dead” on one VAS, ranging from 0 to 100, anchored in the best and worst imaginable health state (Appendix 2). Two participants had to be excluded due to missing data in this task. Sintonen performed a similar task in the original Finnish 15D valuation study but did not use the value of the worst possible 15D health state when finally estimating the Finnish 15D value algorithm [7].

### Empirical performance of the Norwegian 15D health state values

To demonstrate the properties of the Norwegian 15D health state values, we used self-reported health in the Norwegian MIC data set (*N* = 1177) [4, 16]. Details about the data collection were described elsewhere [4, 16]. Respondents filled in the descriptive systems of AQoL-8D [17], EQ-5D-5L [18], HUI 3 [19], and SF-36v2 to derive SF-6D [20], and the 15D [2]. We visually compared the mean health state values of the different instruments in a healthy sample and in seven different disease groups, all collected in Norway. The existing value sets were used to estimate health state values per instrument for a healthy sample and seven disease groups: asthma, cancer, depression and anxiety, diabetes, hearing disability, arthritis, and heart disease. Note that except for the Norwegian 15D value algorithms, none of the other instruments had a value set that is based on preferences of the Norwegian general population. We applied both the Norwegian 15D value algorithm estimated in this study and the one estimated earlier [9] to self-reported health, using the 15D descriptive system, in the Norwegian MIC data set.

## Results

### Descriptive statistics

Table 2 provides an overview of the characteristics of the Norwegian general population in 2010 and the unweighted Norwegian valuation study sample. Responses were weighted using post-stratification to better reflect the makeup of the Norwegian adult general population [21]. Weights were calculated separately for each of the 15 within dimension tasks, and for the pits task. Respondents were categorized by age, gender, and education, and weights were calculated by dividing the proportion of the general population in each category by the corresponding proportion of the respondent sample (see Appendix 3 for details).

**Table 2:** Characteristics of the Norwegian general population in 2010 and the unweighted Norwegian 15D valuation sample

	Norwegian population (*n* = 3,937,847)	Unweighted sample (*n* = 2256)
Gender		
Men	1,956,835 (50%)	1089 (48%)
Women	1,981,012 (50%)	1167 (52%)
Age		
18–24^a^	575,921 (15%)	140 (6%)
25–39	985,937 (25%)	433 (19%)
40–59	1,331,512 (34%)	895 (40%)
60–66	398,529 (10%)	360 (16%)
Older than 67	645,948 (16%)	428 (19%)
Education		
Elementary school	1,111,379 (28%)	472 (20%)
High school	1,625,640 (41%)	960 (43%)
University B.A.	811,360 (21%)	449 (20%)
University M.A.	269,627 (7%)	318 (14%)
No formal education	119,841 (3%)	57 (3%)

^a^The Norwegian sample data include individuals from the age of 18, while the Norwegian population data include individuals from the age of 16

Of 1936 individuals, 1003 finished the Norwegian 15D valuation survey in the Web sample (response rate 52%), while 1276 out of 4899 contacted individuals in the postal sample returned completed surveys (response rate 26%). An overview of the excluded participants can be found in Appendix 5. Participants were aged between 19 and 101 years (mean = 51.6, SD = 16.5), 52% were female, and the most common educational degree was a high school degree (43%). The sample largely matched the underlying population’s characteristics, although responders of the postal survey tend to be older and better educated. Of 118 individuals who provided complete data in the pits-task, 52 participants assigned a higher (“better”) score to “being dead” than to the worst possible 15D health state. While 48 participants chose the same score for “being dead” and the worst possible 15D health state, namely zero.

### The Norwegian 15D value algorithm

In terms of disutility values, the Norwegian 15D health state values ranged from 0 to 1.52, being anchored in an empirical estimate of the worst possible 15D health state.

A Norwegian 15D health state value can be computed with the following formula:

$${\widehat{V}_{H~}}\left( \varvec{l} \right)\mathop =\limits^{{{\text{def}}}} 1 - \frac{{{{\widehat{V}}_{{\text{Pits}}}}}}{{\mathop \sum \nolimits_{j} {{\widehat {S}}_{j,5}}}}~\mathop \sum \limits_{{1 \leqslant j \leqslant 15}} {\widehat {S}_{j,{l_j}}}=1 - \mathop \sum \limits_{{1 \leqslant j \leqslant 15}} 0.113 \cdot ~{\widehat {S}_{j,{l_j}}}=1 - \mathop \sum \limits_{{1 \leqslant j \leqslant 15}} {\widehat {T}_{j,{l_j}}}~$$

The values for ${\widehat {S}_{j,i}}$ can be found in Table 7 in Appendix 4 and the values for ${\widehat {T}_{j,i}}~$ are shown in Table 1 (see also Appendix 4).

**Fig. 2:**
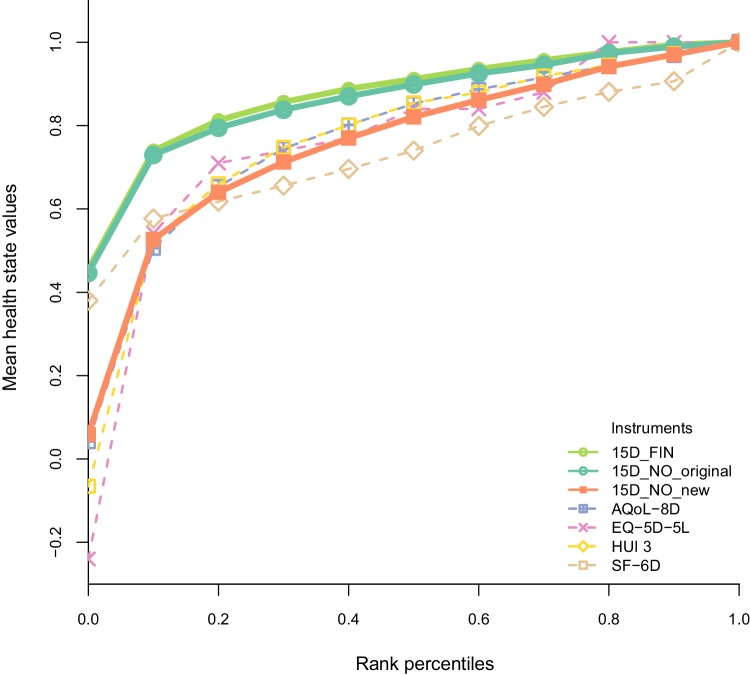
Norwegian 15D health state values compared with health state values of other generic preference-based instruments. Mean health state values by ranked percentiles. *15D_FIN* Finnish 15D algorithm values were calculated with the original procedure ([7], scoring sheet available from Harri Sintonen), *15D_NO_original* Norwegian 15D algorithm values were calculated with the original procedure (Table 3 in [9]), *15D_NO_new* Norwegian 15D algorithm values were calculated with the new procedure

**Fig. 3:**
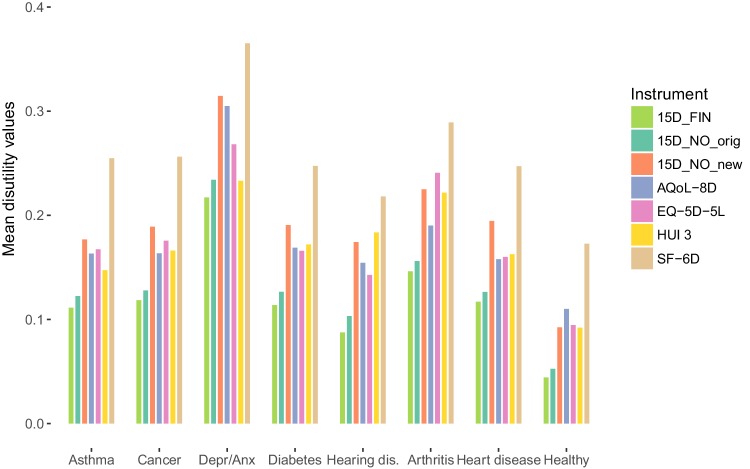
Mean disutility values per disease group and per instrument. *Depr/Anx* depression and anxiety, *hearing dis*. hearing disability, *15D_FIN* Finnish 15D disutility values were calculated with the original procedure ([7], scoring sheet available from Harri Sintonen), *15D_NO_original* Norwegian 15D disutility values were calculated with the original procedure (Table 3 in [9]), *15D_NO_new* Norwegian 15D disutility values were calculated with the new procedure

**Fig. 4:**
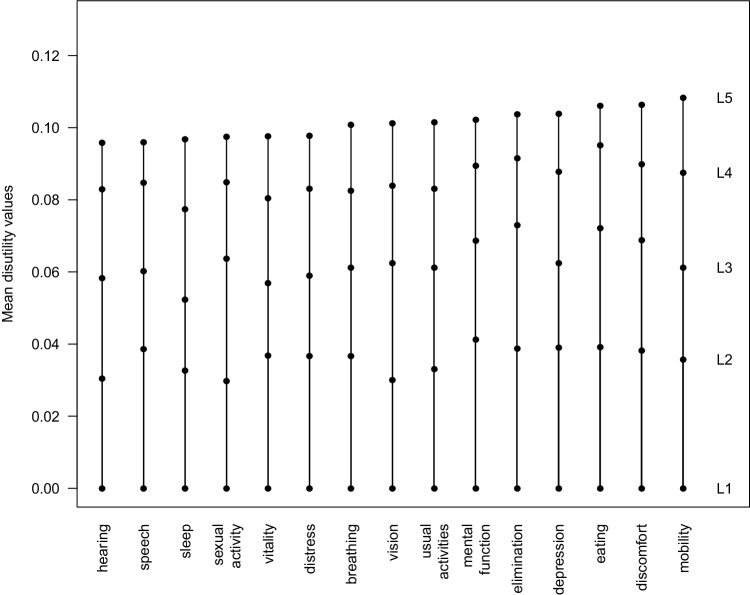
Norwegian disutility algorithm values. Dimensions are ordered by increasing level 5 disutility values of the Norwegian valuation sample. As disutility values are presented, level 1 has a disutility value of zero and level 5 has the largest disutility value

The 15D health state values resulting from using the new procedure are lower than the original 15D values (Figs. 2, 3). The dimensions with the largest L5 disutility values were mobility (0.1083), discomfort (0.1063), and eating (0.1061), whereas hearing (0.0959), speech (0.0960), and sleep (0.0968) had the smallest disutility values on the worst level of function (Table 1; Fig. 4).

### Empirical performance of the Norwegian 15D health state values

In accordance with all other instruments, the largest mean health state disutility value using the Norwegian 15D value algorithm was found for the disease group Depression and Anxiety (Fig. 3). Compared to the original 15D values, the new Norwegian values are in general more in line with corresponding values of other instruments in seven disease groups and a healthy sample (Fig. 3). The same is true when assessing the ranges of the value algorithms (Fig. 2).

## Discussion

### Summary of results

We estimated a Norwegian 15D value algorithm using a new value algorithm estimation procedure. Compared to the 15D health state values that were calculated with the original valuation procedure, the Norwegian 15D health state values were lower and closer to corresponding values of other generic preference-based instruments. With this study, we aimed to amend the available information on the 15D valuation procedure by increasing transparency and replicability of the valuation procedure used to estimate 15D health state values. We aimed to facilitate future research that empirically compares health state values resulting from different instruments, as well as enabling instrument users and decision makers to make more informed choices between generic preference-based instruments.

### The new 15D value algorithm estimation procedure

The new 15D value algorithm estimation procedure provides a more transparent link between the valuation tasks and the resulting 15D health state values. Using fewer valuation tasks reduces the burden and costs related to data collection. Anchoring the 15D algorithm values in an empirically assessed value for the worst possible 15D health state has several advantages. First, this is the most direct way to determine the range of health state values for the instrument. The range is a crucial feature of an instrument. Anchoring the range in an empirical value for the worst possible health state increases the range’s validity compared to the original value range which was based on extrapolation from single dimensions. Second, as the value for the worst possible health state is assessed in relation to “being dead,” the resulting health state values fulfill the requirement of the QALY model better than the original 15D health state values as they are on a ratio-scale with a clearly defined zero. Third, the value of the worst possible health state is the only 15D health state that potentially captures interactions between the levels. This is a valuable amendment to the within dimension tasks scores, which were assessed for each dimension separately.

The empirical value for the worst possible 15D health state assessed in the Norwegian general population is comparable to a similar Finnish value (− .334) [7]. However, this estimate was not used in the Finnish valuation study. The anchoring approach chosen in the new 15D valuation procedure is not without alternatives. Although it possibly is very challenging to value the worst possible 15D health state, having all dimensions at the same worst level of function might still be easier to conceptualize than a health state including different levels of function. Given that it seems unfeasible to value 15D health states that contain different levels of function, we argue that the estimate of the worst possible 15D health state is the most suitable score for anchoring, given the 15D specific constraints due to its large number of health states. One alternative approach would have been to anchor the values in the sum of all L5 disutility values. We did not use this approach as it would have resulted in an unacceptable health state value-range of 0–14.4 in terms of disutility values.

### Empirical performance of the Norwegian 15D health state values

The Norwegian 15D health state values are lower than the original 15D values due to a lower value that was assigned to the worst possible 15D health state. As a consequence, Norwegian 15D health state values are more in line with the values of other generic preference-based instruments (Fig. 2). This will reduce the chance for drawing different conclusions about the cost-effectiveness of health interventions due to using different instruments to assess HRQoL for QALY calculations. As there is no gold standard that indicates if health state values are valid representations of people’s preferences for health, we assessed convergent validity by comparing Norwegian 15D health state values to values of other HRQoL instruments [22]. Compared to the original 15D values, the new Norwegian 15D health state values are closer to the values of other generic preference-based instruments in a healthy subsample as well as in seven disease groups. As we do not know to what extent the health state values of the other instruments are valid representations of people’s preferences, conclusions about validity remain preliminary.

### Remaining challenges and limitations

The original 15D valuation system has been criticized on several grounds. A widely hold criticism is that VAS is used as a stand-alone valuation method [23–26]. It has been questioned if VAS can assess respondent’s preferences as the resulting valuations are not the result of a trade-off [27, 28]. A potential concern with the new 15D valuation procedure is that it relies on trade-offs between the levels of different dimensions compared to death to determine the relative weight of dimensions, rather than direct trade-offs between dimensions. The primary strength of this method over direct trade-offs between dimensions is that it reduces the cognitive burden of the tasks, allowing respondents to focus on the impact of each specific level of each dimension compared to death. However, the absence of direct trade-offs could attenuate differences between dimensions. There are also a number of biases related to VAS that might influence health state valuation [23, 29]. However, the VAS has been defended as a preference elicitation method for eliciting health state values under certainty [30] and valuation methods that are similar to those of the 15D have been recently applied in a new method for valuing health [31]. Another aspect that has been criticized is that an additive model is used to estimate 15D health state values. The additive model has been chosen by the 15D instrument developer as other models have not been considered to be feasible due to the number of potential interactions [7]. Using an additive model means to assume that all dimensions are structurally independent. To our knowledge, the structural independence of the 15D dimensions has not been tested.

While this study identified and addressed a selection of methodological shortcomings of the 15D valuation system, others remain to be addressed in future research. Although we were aware of VAS’ shortcomings, we kept using VAS as a valuation method in the new 15D valuation procedure. The use of other valuation methods, or allowing trade-offs between the dimensions, is complicated by the large descriptive system of the 15D. The advantage of keeping the same valuation method as in the original valuation procedure is that already existing 15D value algorithms can be converted into health state values that are more in line with requirements of the QALY model. This can be done by assessing an empirical value for the worst possible 15D health state and anchoring the already existing 15D algorithm values in this pits-estimate. In these kind of studies, the estimate of the worst possible 15D health state could also be assessed with other valuation methods than VAS. However, it has to be kept in mind that valuing a health state with 15 attributes is challenging, especially when assessing worse than death values [32]. Furthermore, we kept using the additive model in the new 15D procedure. It is unsatisfying that no explicit tests have been performed to empirically support this model choice. However, testing the structural independence of the 15 domains and assessing the interactions between them are extensive tasks which were beyond the scope of this study. Additionally, we recommend that future studies use identical task presentations of the within dimension tasks in the Web and the postal sample. Finally, it is worth noticing that the disease classification in the MIC data set was based on self-reports rather than on medical diagnosis. This may limit the clinical accuracy of these groups. For the purpose of comparing different instruments, however, this is not a central concern.

## Conclusions

The Norwegian 15D value algorithm is the result of applying a new 15D valuation procedure that uses fewer valuation tasks and is anchored in an empirically assessed value for the worst possible 15D health state. The Norwegian 15D health state values are more in line with the requirements of the QALY model and are more comparable to the values of other HRQoL instruments in seven disease groups and a healthy sample. This study presents the first Norwegian value set for a generic preference-based instrument. We recommend using the new 15D valuation procedure when estimating future 15D value algorithms in general, and the Norwegian 15D health state values specifically for 15D-based health economic analyses in Norway.

## Appendix 1: Task presentation of the within dimension tasks in the postal and the Web subsample

In the postal sample, a vertical VAS was used, with L1 being fixed to 100 (see “Visual analogue scale used for the within dimension tasks in the postal sample”). In the Web sample, a horizontal VAS was used, and the indicators for L1 were not fixed (see “Vertical visual analogue scale used for the within dimension tasks in the Web sample”). In order to improve comparability with the postal sample, where L1 was fixed to 100, we rescaled the cases of the Web sample to 100 if the VAS-score assigned to L1 was less than 90.

## Appendix 2: Instruction and task presentation of the pits-task

“In the following task, two health states are described. We want you to compare these health states with each other. First, read the entire description of both health states. Then, we kindly ask you to first assign a number to the health state that you consider to be worst and then to the remaining health state. You can assign all numbers between 0 and 100 as you consider it to be most suitable.”

## Appendix 3: Post-stratification weighting

The aim of applying weights to the valuation data was to make the valuation sample representative of the Norwegian general population at the time of data collection. We applied a weighting method referred to as “post*-*stratification weighting” in the literature [21].

The participants of the Norwegian 15D valuation study were randomized to perform different subsets 15D valuation tasks (for details see [9]). As also case exclusions were performed separately by task (see Appendix 5 for details), the precise makeup of respondents varied by task. Therefore we estimated 16 task-specific sets of weights: one for each of the fifteen within dimension tasks, and one for the pits-task.

The following describes the technical details of how we estimated the weights for post*-*stratification. First, we assigned each member of the Norwegian general population to categories that were defined by different combinations of age, gender (and education) (see Tables 3, 4 in Appendix 3). The respondents of the Norwegian valuation studies have been assigned to the same categories. The weight for the respondents of one category is defined by the proportion of the category in the population divided by the proportion of the same category in the sample (see Table 5 in Appendix 3).

For additional clarity, a specific example of how the weight of one category was estimated follows. In this example we estimated weights for the *within dimension task* for the mobility dimension. Consider the category included all respondents who were female, aged 30–39, with education equivalent to BA level. This group was represented by 122,516 individuals in the Norwegian general population, and by 67 persons in the respondent sample that performed the *within mobility task*. The mobility-specific weight for this category is estimated by dividing the proportion in the population by the corresponding proportion in the mobility-specific subsample, i.e.:

$$\frac{{(122516/3937847)}}{{(67/424)}}~=~0.1968906$$

The population proportions were retrieved from Statistics Norway (http://www.ssb.no). We used the population makeup from the year of study, i.e. 2010 for the within dimension task weights, and 2016 for the pits-state weights. The variable coding used to define the stratification categories can be found in Tables 3 and 4 in Appendix 3.

Table 5 in Appendix 3 provides weights for the pooled sample of the Norwegian valuation study. We also included the proportions of each category in the population and the pooled Norwegian 15D valuation sample respectively. Each cell of Table 5 in Appendix 3 contains the following information:

$$\frac{{{\text{Proportion~of~the~respective~category~in~the~Norwegian~general~population~}}({\text{in}}~\% ~)}}{{~~~~{\text{Proportion~of~the~~respective~category~in~the~pooled~Norwegian~valuation~sample~}}({\text{in}}~\% )}}={\text{Pooled~sample~weight~of~respective~category}}$$

**Table 3:** Coding of the variables used to estimate weights in the Norwegian 15D valuation study

Gender	
M	Men
F	Women
Age	
AGE1	18–19
AGE2	20–24
AGE3	25–29
AGE4	30–39
AGE5	40–49
AGE6	50–59
AGE7	60–66
AGE8	Older than 67
Education	
EDU1	Elementary school
EDU2	High school
EDU3	University B.A.
EDU4	University M.A.
EDU5	No formal education

**Table 4:** Coding of the variables used to estimate weights in the pits-data collection

Gender	
M	Men
F	Women
Age	
AGE1	16–29
AGE2	30–49
AGE3	Older than 50

**Table 5:** Proportions and weights of each category for the pooled Norwegian 15D valuation sample

	EDU1	EDU2	EDU3	EDU4	EDU5
AGE1_M^a^	$$\frac{{2.94526\%}}{{0.39894\%}}$$ = 7.38271	$$\frac{{0.33021\%}}{{0.39894\%}}$$ = 0.827718	–^b^	–^b^	–^b^
AGE2_M	$$\frac{{1.3707\%}}{{0.1773\% }}~$$ = 7.73096	$$\frac{{2.1128\%}}{{1.46277\% }}~$$ = 1.44438	$$\frac{{0.42914\%}}{{0.04433\%}}$$ = 9.68058	$$\frac{{0.01331\%}}{{0.04433\% }}$$ = 0.300248	$$\frac{{0.17588{\text{\%}}}}{{0.04433{\text{\% }}}}$$ = 396752$$
AGE3_M	$$\frac{{3.541\% ~}}{{0.26596\% }}$$ = 3.54147	$$\frac{{1.55146\% ~}}{{0.8422\% }}~$$ = 1.84215	$$\frac{{0.89298\% ~}}{{0.62057\% }}$$ = 1.43897	$$\frac{{0.33221\% ~}}{{0.26596\% }}$$ = 1.2491	$$\frac{{0.28899{\text{\% }}}}{{0.04433{\text{\% }}}}$$ = 6.51906
AGE4_M	$$\frac{{1.50476\% ~}}{{0.66489\% }}$$ = 2.26317	$$\frac{{3.72554\% ~}}{{2.65957\% }}~$$ = 1.40081	$$\frac{{1.98558{\text{\% }}}}{{1.46277{\text{\% }}}}$$ = 1.35741	$$\frac{{1.04395{\text{\%}}}}{{1.50709{\text{\% }}}}$$ = 0.692693	$$\frac{{0.49702{\text{\% }}}}{{0.04433{\text{\% }}}}$$ = 11.2118
AGE5_M	$$\frac{{1.96021{\text{\% }}}}{{1.72872{\text{\% }}}}$$ = 1.13391	$$\frac{{4.25222\% ~}}{{4.52128\% }}$$ = 0.94049	$$\frac{{1.86086{\text{\% }}}}{{1.55142{\text{\% }}}}$$ = 1.19946	$$\frac{{0.90136{\text{\% }}}}{{1.46277{\text{\% }}}}$$ = 0.616201	$$\frac{{0.30974{\text{\% }}}}{{0.04433{\text{\% }}}}$$ = 6.98714
AGE6_M	$$\frac{{0.674{\text{\% }}}}{{2.43794{\text{\% }}}}$$ = 0.674036	$$\frac{{3.95104\% ~}}{{4.3883\% }}$$ = 0.900358	$$\frac{{0.771{\text{\%}}}}{{1.95035{\text{\% }}}}$$ = 0.771026	$$\frac{{0.7479{\text{\% }}}}{{1.46277{\text{\% }}}}$$ = 0.51129	$$\frac{{0.16461{\text{\% }}}}{{0.08865{\text{\% }}}}$$ = 1.85685
AGE7_M	$$\frac{{1.07338{\text{\%}}}}{{1.90603{\text{\% }}}}~$$ = 0.56315	$$\frac{{2.57727{\text{\% }}}}{{3.32447{\text{\% }}}}$$ = 0.775242	$$\frac{{0.8932{\text{\%}}}}{{1.0195{\text{\% }}}}$$ = 0.876116	$$\frac{{0.49837{\text{\%}}}}{{1.10816{\text{\% }}}}$$ = 0.449727	$$\frac{{0.05442{\text{\% }}}}{{0.35461{\text{\% }}}}$$ = 0.153464
AGE8_M	$$\frac{{2.30651{\text{\% }}}}{{2.21631{\text{\% }}}}~$$ = 1.0407	$$\frac{{3.26615{\text{\% }}}}{{4.69858{\text{\% }}}}$$ = 0.695136	$$\frac{{0.867{\text{\% }}}}{{1.19681{\text{\% }}}}$$ = 0.724426	$$\frac{{0.53065{\text{\% }}}}{{1.37411{\text{\% }}}}$$ = 0.386177	$$\frac{{0.07408{\text{\% }}}}{{0.48759{\text{\% }}}}$$ = 0.151931
AGE1_F	$$\frac{{2.6385\% }}{{0.53191\% }}$$ = 4.96043	$$\frac{{0.45924\% }}{{0.35461\% }}$$ = 1.29506	–^b^	–^b^	–^b^
AGE2_F	$$\frac{{0.9672{\text{\% }}}}{{0.35461{\text{\% }}}}$$ = 2.7275	$$\frac{{1.9325{\text{\% }}}}{{1.72872{\text{\% }}}}$$ = 1.11788	$$\frac{{0.83838{\text{\% }}}}{{0.62057{\text{\% }}}}$$ = 1.35098	–^b^	$$\frac{{0.19853{\text{\% }}}}{{0.04433{\text{\% }}}}$$ = 4.47846
AGE3_F	$$\frac{{0.66572{\text{\% }}}}{{0.13298{\text{\% }}}}$$ = 5.00617	$$\frac{{1.09314{\text{\% }}}}{{0.93085{\text{\% }}}}$$ = 1.17435	$$\frac{{1.47631{\text{\% }}}}{{0.97518{\text{\% }}}}$$ = 151388	$$\frac{{0.38706{\text{\% }}}}{{0.75355{\text{\% }}}}$$ = 0.513649	$$\frac{{0.2703{\text{\% }}}}{{0.04433{\text{\% }}}}$$ = 6.09745
AGE4_F	$$\frac{{1.24215\% }}{{0.88652\% }}$$ = 1.40115	$$\frac{{2.65561\% }}{{2.26064\% }}$$ = 1.17472	$$\frac{{3.11124\% }}{{2.96986\% }}$$ = 1.0476	$$\frac{{1.01652\% }}{{1.72872\% }}$$ = 0.588019	$$\frac{{0.35504\% }}{{0.13298\% }}$$ = 2.66988
AGE5_F	$$\frac{{1.82414\% }}{{1.59574\% }}$$ = 1.14313	$$\frac{{3.36793\% }}{{3.5461\% }}$$ = 0.949756	$$\frac{{2.7189\% }}{{2.92553\% }}$$ = 0.92937	$$\frac{{0.68388\% }}{{1.37411\% }}$$ = 0.497689	$$\frac{{0.18495\% }}{{0.13298\% }}$$ = 1.39081
AGE6_F	$$\frac{{1.80784\% }}{{2.26064\% }}$$ = 0.799703	$$\frac{{3.40186\% }}{{4.16667\% }}$$ = 0.816446	$$\frac{{2.02743\% }}{{2.08333\% }}$$ = 0.973168	$$\frac{{0.38963\% }}{{1.64007\% }}$$ = 0.237569	$$\frac{{0.11169\% }}{{0.31028\% }}$$ = 0.359965
AGE7_F	$$\frac{{1.21823\% }}{{2.30496\% }}$$ = 0.528525	$$\frac{{2.60584{\text{\% }}}}{{3.41312{\text{\% }}}}$$ = 0.763477	$$\frac{{0.98132\% }}{{1.37411\% }}$$ = 0.71415	$$\frac{{0.16093\% }}{{0.93085\% }}$$ = 0.172885	$$\frac{{0.05752\% }}{{0.22163\% }}$$ = 0.259532
AGE8_F	$$\frac{{4.11326\%}}{{3.05851\% }}$$ = 1.34486	$$\frac{{3.99965\% }}{{3.85638\% }}$$ = 1.03715	$$\frac{{1.01754\% }}{{1.10816\% }}$$ = 0.918225	$$\frac{{0.12273\% }}{{0.44326\% }}$$ = 0.27688	$$\frac{{0.10602\% }}{{0.53191\% }}$$ = 0.199319

*AGE* age group, *EDU* level of education, *F* female, *M* male

^a^AGE1_M refers to males (M) who are aged 18–19 (AGE1), see Tables 3, 4 in Appendix 3 for the coding of the variables

^b^Some categories are empty (–) as there are no individuals of so young age with a higher educational degree

## Appendix 4: Flow chart and data inputs of the new value algorithm estimation procedure

The following figure provides an overview of the 15D valuation system. The figure links the terms used in the step-by-step description of the new valuation procedure to the data presented in Tables 6 and 7 in Appendix 4.

**Table 6:** Weighted mean VAS-scores

	Within dimension task scores					
	L1	L2	L3	L4	L5	Death
Hearing	100	75.64	53.34	33.59	23.24	9.31
Speech	100	68.56	50.91	31.01	21.83	7.73
Sleep	100	73.97	58.31	38.42	22.99	9.85
Sexual activity	100	76.28	49.14	32.22	22.20	9.59
Vitality	100	71.34	55.78	37.46	24.18	11.96
Distress	100	71.35	54.01	35.15	23.70	11.63
Breathing	100	69.89	49.77	32.30	17.25	7.01
Vision	100	76.33	50.76	33.84	20.20	10.70
Usual activities	100	73.92	51.83	34.59	20.05	10.83
Mental function	100	67.15	45.31	28.82	18.64	9.86
Elimination	100	69.10	41.94	27.14	17.36	9.78
Depression	100	69.57	51.26	31.57	19.00	11.67
Eating	100	67.94	41.03	22.18	13.23	7.36
Discomfort	100	70.79	47.33	31.28	18.73	13.38
Mobility	100	70.84	50.03	28.46	11.52	7.47

With the scores in Table 6${\widehat {S}_{j,i}}$ can be estimated. *j* corresponds to dimensions and can be found in the rows of Table 6, while *i* corresponds to the levels which can by found in the columns of Table 6. For example, the weighted and averaged VAS scores for level four of the hearing dimension (${\widehat {S}_{1,4}}$) can be found in row 1, column 4 of Table 6

**Table 7:** Rescaled within dimension disutility scores

	L1	L2	L3	L4	L5
Hearing	0.0000	0.2686	0.514	0.7323	0.8464
Speech	0.0000	0.3408	0.5321	0.7478	0.8472
Sleep	0.0000	0.2887	0.4624	0.6831	0.8543
Sexual activity	0.0000	0.2623	0.5625	0.7497	0.8605
Vitality	0.0000	0.3255	0.5023	0.7104	0.8612
Distress	0.0000	0.3242	0.5204	0.7339	0.8633
Breathing	0.0000	0.3238	0.5402	0.7280	0.8899
Vision	0.0000	0.2651	0.5514	0.7409	0.8937
Usual activities	0.0000	0.2925	0.5403	0.7336	0.8966
Mental function	0.0000	0.3644	0.6067	0.7896	0.9025
Elimination	0.0000	0.3425	0.6436	0.8076	0.9159
Depression	0.0000	0.3444	0.5518	0.7747	0.9170
Eating	0.0000	0.3461	0.6365	0.8400	0.9366
Discomfort	0.0000	0.3372	0.6080	0.7934	0.9382
Mobility	0.0000	0.3151	0.5400	0.7731	0.9562

With the scores in Table 7${\widehat {T}_{j,i}}$ can be estimated.* j* corresponds to dimensions and can be found in the rows of Table 7, while *i* corresponds to the levels with can by found in the columns of Table 7. For example, the rescaled dimension disutility score for level four of the hearing dimension (${\widehat {T}_{1,4}}$) can be found in row 1, column 4 of Table 7

## Appendix 5: Cases excluded in the Norwegian valuation study

**Table:**

Dimension	Exclusions by criteria			
	Finally excluded^a^	Value lower than 90 on level 1	2 or more missing on levels 2 through 5	Same value to levels 2 through 5
Hearing	140	9	100	38
Speech	161	2	109	50
Sleep	129	12	96	25
Sexual activity	149	12	112	29
Vitality	129	12	88	29
Distress	126	7	90	31
Breathing	136	3	96	37
Vision	139	3	95	43
Usual activities	148	9	101	41
Mental function	142	5	99	40
Elimination	159	14	105	42
Depression	143	10	104	33
Eating	154	1	98	56
Discomfort	142	16	98	30
Mobility	146	8	103	36

^a^There is some overlap of excluded cases. Therefore, the sum of all three exclusion criteria does not correspond to the number of finally excluded
